# A history of futures: A review of scenario use in water policy studies in the Netherlands

**DOI:** 10.1016/j.envsci.2012.03.002

**Published:** 2012-05

**Authors:** M. Haasnoot, H. Middelkoop

**Affiliations:** a Deltares, P.O. Box 177, 2600 MH Delft, The Netherlands; b Utrecht University, Department of Physical Geography, P.O. Box 80115, 3508 TC Utrecht, The Netherlands; c University of Twente, Department of Water Engineering and Management, P.O. Box 217, 7500 AE Enschede, The Netherlands

**Keywords:** Scenarios, Water management, Climate change, Uncertainty, The Netherlands

## Abstract

The future of human life in the world's river deltas depends on the success of water management. To deal with uncertainties about the future, policymakers in the Netherlands have used scenarios to develop water management strategies for the coastal zone of the Rhine–Meuse delta. In this paper we reflect on six decades of scenario use in the Netherlands, and provide recommendations for future studies. Based on two criteria, ‘Decision robustness’ and ‘Learning success’, we conclude that (1) the possibilities for robust decisionmaking increased through a paradigm shift from predicting to exploring futures, but the scenario method is not yet fully exploited for decisionmaking under uncertainty; and (2) the scenarios enabled learning about possible impacts of developments and effectiveness of policy options. New scenario approaches are emerging to deal with the deep uncertainties water managers are currently facing.

## Graphical abstract

**Graphical abstract:**

**Figure:**
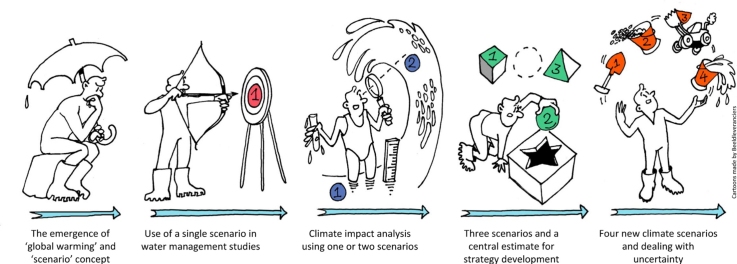

**Highlights:**

► We reflect on 6 decades of scenarios use in Dutch water management studies. ► We observed a paradigm shift from predicting to exploring the future. ► This increased the opportunity for robust decision-making. ► The scenarios enabled learning about impacts and effectiveness of policy options and raised awareness. ► The turn-over rate from science to policy has speeded-up and further intensified.

## 1 Introduction

The world's river deltas are increasingly vulnerable due to pressures from climate change, relative sea level rise and population growth (Syvitski et al., 2009; Vörösmarty, 2009). Therefore, densely populated deltas such as the Netherlands require well-designed water management for flood protection and for coping with varying water demands and availability.

Water management decisions should bring solutions that will sustain for several decades, implying that they should be adequate even in case of changes in pressures. However, uncertainties about the future make decisionmaking less straightforward. Therefore, policymakers increasingly use *robustness* as indicator in decisionmaking. A robust strategy performs relatively well across wide range of possible futures (Lempert et al., 2006) and other uncertainties. Water management faces uncertainties arising from (1) natural uncertainties such as trends and extreme weather events; (2) social uncertainties due to shifts in human response and values; (3) technological uncertainties through modelling future states and impact (e.g. Haasnoot et al., 2011).

Scenario analysis is a method for dealing with uncertainties, and aims to assess possible impacts and to design policies (e.g. Carter et al., 2007). Scenarios are coherent descriptions of alternative hypothetical futures that reflect different perspectives on past, present and future developments, which can serve as a basis for action (Van Notten, 2005). Since its first use in military planning in the 1950s (Bradfield et al., 2005; Brown, 1968; Kahn and Wiener, 1967), scenario analysis has been applied in a variety of areas, such as business development (Bradfield et al., 2005; Van der Heijden, 1996; Wack, 1985), environmental planning (Alcamo, 2009, 2001; Peterson et al., 2003) and climate change mitigation and adaptation (Hulme and Dessai, 2008; IPCC, 2000; Rosentrater, 2010; Wigley et al., 1980). Scenarios have also been used for robust decisionmaking in case of complex problems with deep uncertainty, such as long-term water management under changing conditions (e.g. Lempert and Schlesinger, 2000; Dewar et al., 1993; Lempert and Bankes, 2003; Lempert et al., 2006; Groves, 2006; Kwakkel et al., 2010 or Middelkoop et al., 2004; Van Asselt and Rotmans, 2002; Dessai and Hulme, 2007 for examples related to water management).

To enable life in a low-lying delta, the Dutch have had a long history of controlling and maintaining the water system. In the Netherlands, scenarios have been used since the 1950s to prepare water management for the future. After six decades of experience, we reflect on scenario use in water management in the Netherlands, and identify possible improvements for future studies. This evaluation provides more insight in policymaking on water management in river deltas under uncertainty to support the current development of the next generation scenarios for climate adaptation studies.

This paper provides a review of scenario use in water management studies on the Rhine–Meuse delta in the Netherlands, and evaluates the lessons that can be derived from this experience. We seek to answer the following questions: What was the evolvement of scenario use in water management? Did the scenarios provide prospect for robust decisionmaking? Did the scenarios enable learning for policymakers and/or scientists? After giving a historical perspective, we evaluate the scenario use based on two criteria: ‘Decision robustness’ and ‘Learning success’. We end the paper with conclusions and recommendations for future water management studies.

## 2 Approach for evaluating the scenario use

**Fig. 1:**
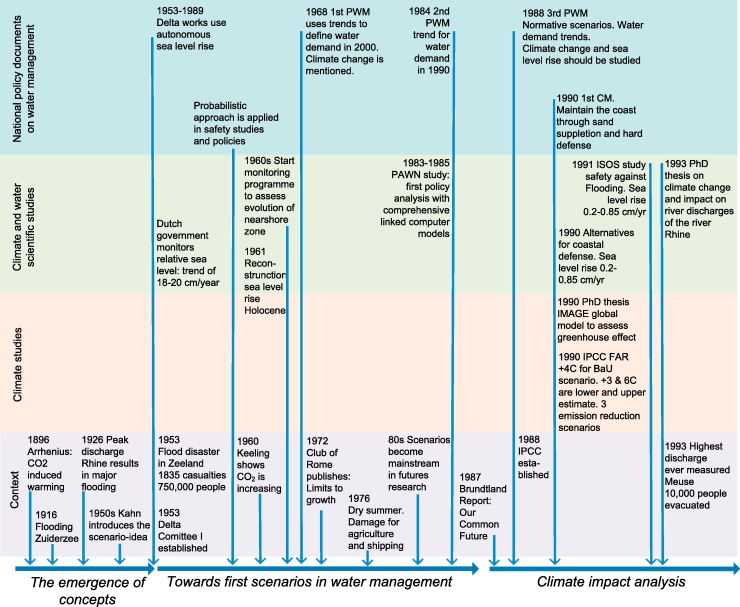

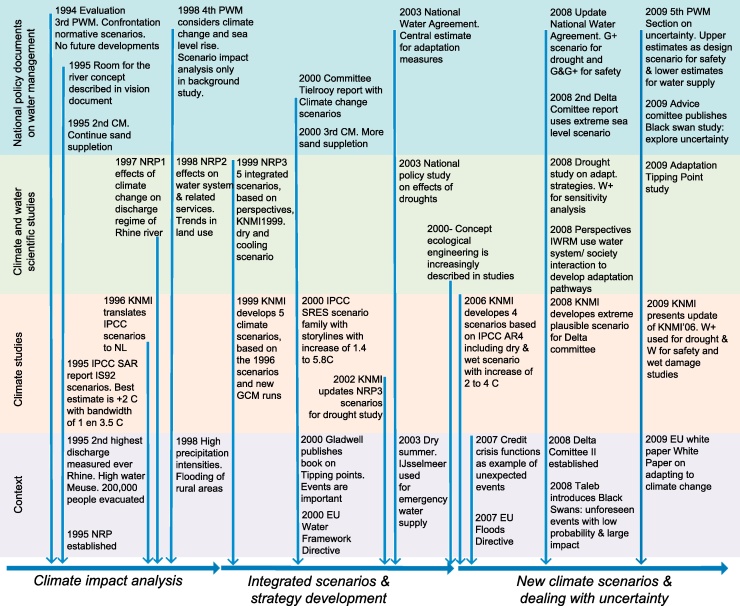
Historical perspective on developments in national water policy documents in the Netherlands, key research studies on climate and water, climate scenario studies and the context in which these studies were made. PWM = National Policy Memorandum on Water Management; CM = Coastal Memorandum.

For our chronology on scenario use in water management in the Netherlands we reviewed all national water policy documents, the key research studies on climate and water, and related climate scenario studies. In addition, we used our own experience, based on participation in several water policy studies since the 1990s, and the experience of several colleagues, who were involved in earlier water policy studies or climate scenario studies. We present the studies from the Netherlands against the (inter)national context (see Fig. 1 for overview and supplementary information for more characteristics).

For our analysis we adopted two criteria used by Hulme and Dessai (2008b) in a framework for climate scenario evaluation, which we further refer to as the ‘Decision robustness’ and the ‘Learning success’.

The ‘Decision robustness’ criterion can be addressed with the following question: *‘do the scenarios contain a sufficient representation of relevant knowable uncertainties to offer the prospect that decisions taken with support of the scenarios will be robust?’* Robustness is an important criterion for good decisions under uncertainty (Rosenhead et al., 1972; Metz et al., 2001), especially by policymakers facing deep uncertainty (Lempert et al., 2006; Groves and Lempert, 2007). By including uncertainties in decisionmaking it is possible to identify strategies that perform relatively well under various different possible futures (robust strategies), or to make a well-thought-out decision on whether or not to adapt a strategy in view of a specific uncertainty. Assessing the robustness of decisions is relevant, because decisions involve large high-cost investments, and can have large implications for society. Therefore, water management decisions should be cost-effective for several decades, even if the future turns out to be different from what was anticipated.

Intuitively, one might consider the following question as a criterion for evaluating the ‘Decision robustness’ (in retrospect): *‘was the decision taken a ‘good’ decision?’* However, there are some fundamental problems in answering this question. Firstly, major water management decisions have often a long implementation time, or involve strategies with a considerable life-time (e.g. tens of years). Yet, for many studies the time passed has been too short to decide whether decisions have turned out to be successful. Secondly, and more important, we can only evaluate decisions against the single past we had, which is only one realisation of all possible futures that could have evolved after the decision was taken. For example, due to inherent climate variability and the stochastic nature of the occurrence of extremes, prolonged periods can pass without extreme events, even in the case of climate change. If it was decided that anticipatory strategies were not needed, this decision would have been evaluated as ‘good’, as a result of the fortuitous absence of extreme events. In other – equally likely – realisations of the future, in which some extreme events occurred, this decision would have been judged as ‘bad’. So, judging a decision against a single past does not provide a sound indication of its robustness or potential success; such evaluation requires confronting the result to a range of realisations of the future. In our paper, therefore, we focus on whether the decision process – based on the scenarios considered – provided *prospects for robust decisions*.

Indicators for the ‘Decision robustness’ criterion should, therefore, reflect whether relevant uncertainties are sufficiently represented. *Relevant* uncertainties have significant and distinguished impact on the outcomes, and consequently the decisionmaking (cf. IPCC, 2001). For water management this involves uncertainties in both water *demand* and *availability*. This means that scenarios should include uncertainties in climate, sea level and river discharges, that all affect water availability, as well as uncertainties in socio-economic and social developments (e.g. land use and the accepted flood damage), that determine societal requirements and thus the water demand. A different kind of relevant uncertainty arises from interactions between the water system, society and water management. For example, floods and droughts may raise the need for additional or new measures, or more profoundly, it may influence societal perspective (e.g. how we evaluate system and our expectations of the future), and may trigger a water policy response which may then affect the water system. The resulting water management response will then affect the water system and its future response to extremes. Uncertainty in the policy response further adds to the total uncertainty on the water system in the future. In retrospective, water management in the Netherlands has indeed strongly been driven by both floods (e.g. in 1993 and 1995) and drought events (e.g. the summer of 1976), and socio-economic trends (e.g. increasing valuation of nature and cultural heritage). For robust decisionmaking scenarios should, therefore, consider the dynamic interactions among climate, society and water management as these evolve in the course of time and influence the performance of policy options.

To determine whether uncertainties were *sufficiently* represented for robust decisionmaking, we analysed the *range* and *diversity* of the considered scenarios using the following indicators: the number of scenarios, the variety in the range of outcomes encompassed, the variety in alternatives, and the temporal and dynamic nature of the scenarios. Using the range of a scenario as indicator for ‘Decision robustness’ does not mean that decisionmaking should be based only on the extremes nor that a broader range in itself is better. Instead, several alternative scenarios should be considered that encompass a relevant and plausible range of futures. Alternative scenarios go beyond the frequently used ‘business as usual’ scenarios derived by extrapolation of ongoing trends, and comprise changes in developments in the course of time. Regarding the temporal nature of a scenarios, scenarios can be ‘snapshots’ describing a moment in the future, or ‘transient’ scenarios describing the evolvement to a certain point in the future (Van Notten, 2005). The dynamic nature of a scenario refers to whether a scenario is essentially based on a gradual extrapolation of trends, or whether it encompasses events, discontinuities, or even surprises which change gradual developments abruptly (Van Notten, 2005). What is considered ‘plausible’ or ‘relevant’ is subject to different interpretations, and depends on one's expectations about the future and understanding of the system. A way of dealing with this type of uncertainty – often referred to as perspective-based uncertainty – is including such different perspectives in the scenarios (cf. Middelkoop et al., 2004; Van Asselt et al., 9582).

The ‘Learning success’ criterion refers to the question: *did the scenarios enable learning for policymakers and scientists?* Answering this question is relevant to indicate the value of scenario analysis, and to improve future scenario use in water management studies. Although there are many definitions of learning, most theorists agree that learning is a change in knowledge or behaviour as a result of experience (e.g. Kolb, 1984; Driscoll, 1994). Although we could not provide quantitative measures, we determined indications of the learning effect from reflection and underpinnings indicated in the reports. We give some examples: (1) A policy report that mentions results of a scientific long-term water policy study as a starting point of their study (*‘Scenario studies show that climate change will have an impact on the hydrological water system.’*). (2) A policy document mentioning a contextual development or event as a reason to adapt a policy or a scenario (*‘Event x raised awareness that a new scenario/approach is needed.’*). (3) A research study stating that *previous results showed ‘X’, but ‘Y’ is unclear, and will be studied*. Therefore, we analysed the evolution of the scenario content and use, the study's subject, and the science-policy interaction, and use this information in combination with our experience and the experience of our colleagues, to estimate the ‘Learning success’.

## 3 Historical perspective on scenario use in water management studies

### 3.1 The emergence of concepts

The emergence of concept of anthropogenic global warming has been characterised by different milestones (e.g. Peterson et al., 2008; Weart, 2010). Mid-19th century, Tyndall suggested that atmospheric changes could explain ice ages (Tyndall, 1861). Arrhenius was the first to quantify the contribution of CO_2_ to the greenhouse effect (Arrhenius, 1896). In the 1950s, progress in understanding of climate cycles resulted in the Milankovitch theory, explaining cycles at glacial-interglacial time scales (Milankovitch, 1930). After 1950, tools became available for measuring greenhouse gases. Keeling (1960) showed a faster CO_2_ increase than Arrhenius’ estimate. Together with available data on the global temperature this led to the idea that increasing CO_2_ could result in marked climate change (Revelle et al., 1965). In the 1970s, climate models were developed and used to study the combined effect of cooling through aerosols and warming through CO_2_. After warming trends, reported in the 1940s, a multidecade cooling was observed (Mitchell, 1963). Although scientific articles described both potential future warming and cooling, the media (e.g. Gwynne, 1975) mainly covered a future cooler world (Peterson et al., 2008). In the mid-1970s, the discussion in the media became dichotomous: the climate could become warmer or cooler (Mathews, 1976).

The scenario concept originates from the 1950s and is ascribed to Herman Kahn at that time working at the RAND Corporation (Van Asselt et al., 2010). He demonstrated with scenarios that US military planning was based on ‘wishful thinking’ instead of ‘reasonable expectations’ (Bradfield et al., 2005). In the 1970s, scenarios were used to explore the sustainability of natural resources. ‘The limits to growth’ of the Club of Rome is a well-known example (Meadows et al., 1972). Using scenarios and the World3 computermodel the study showed that a long-term perspective can identify problems in current policies (Van Asselt et al., 2010). In business development, Shell Oil is considered the first to use scenario planning (Van der Heijden, 1996; Wack, 1985).

### 3.2 Towards first scenarios in water management (1953–1988)

After a millennium of adaptation in response to (flood) events, the Dutch shifted to anticipatory water management in the course of the twentieth century. The 1916 storm flood along the Zuiderzee initiated the implementation of existing plans for the Afsluitdijk, a large defence structure separating the Zuiderzee from the sea. The 1953 storm surge, which killed 1835 and affected 750,000 people, triggered a paradigm shift. Policymakers learned that the deterministic approach was inadequate. From the perspective that *‘this should never happen again’*, they stated that the probability of occurrence of such an event should be very small. Accordingly, an a-priori accepted exceedance probability and corresponding water level were determined, resulting in design conditions for the Delta Works (Delta Committee, 1960), the large defense structures in the southwest delta. This was the first use of future conditions. A relative sea level rise based on extrapolation of measurements was included in the design of the defense structures, because of its lifetime (100–200 years) (Rijkswaterstaat, 2008). However, a potentially accelerated sea level rise due to climate change was not considered. This probabilistic approach was adopted for all primary flood defences.

Along with the Delta Works the Dutch government decided for developing a national policy on water management, and to document this in a National Policy Memorandum on Water Management (PWM). As safety was ensured with the Delta Works and the Afsluitdijk, the 1st PWM focused on fresh water supply (Rijkswaterstaat, 1968). Although climate change and sea level rise were mentioned, assessments considered only an increase in water demand. Uncertainties about future developments were acknowledged, but no bandwidth was given. The document stated that *‘the influence of these developments (climate change and upstream water use) on the total water availability is considered to be small. It is however important to keep monitoring these developments.’* (Rijkswaterstaat, 1968, p. 137).

In the 1980s, scenarios became mainstream in futures research (Moss et al., 2010). Also, in the Netherlands scenario analysis emerged. This was probably supported by the cooperation with the RAND Corporation for the PAWN-study (Policy Analysis for the Water management of the Netherlands) (RAND Corporation, 1983; Rijkswaterstaat, 1985) that provided the scientific support for the 2nd PWM (Rijkswaterstaat, 1984).

In the 2nd PWM, the government stated that revision of the 1st PWM was needed due to: *‘societal developments, changes in insight and stakeholders of the water system. For example, the prognoses for the future water demands for agriculture and drinking and industry water need to be revised and the importance of sectors like industry, shipping and nature has been acknowledged’* (Rijkswaterstaat, 1984, p. 7). The 2nd PWM emphasised improving water management from a cost-benefit perspective. This was a paradigm shift; instead of ensuring water for all users, policy was now only implemented if the benefits were larger than the costs. Trends in water use were considered for agriculture, drinking and industry water in the policy analysis. The PAWN-study mentions that *‘at places where the uncertainty in the results has an impact on the conclusions, either a sensitivity analysis is executed or different scenarios are described.’* (Rijkswaterstaat, 1985, p. 138). The study concluded that even in case of the ‘maximum trend scenario’ for irrigation, wherein many farmers would use sprinklers, no large interventions were needed. These conclusions were adopted in the 2nd PWM.

### 3.3 Climate change scenarios and impact analysis on the water system (1988–1998)

By the end of the 1980s, experiments with Global Climate Models (GCMs) indicated that the signal of anthropogenic warming would soon emerge from natural variability (Hansen, 1988; Moss et al., 2010). The International Panel on Climate Change (IPCC) published its first assessment including four scenarios in 1990 (IPCC, 1990). The scenario ‘business as usual’ (BaU) assumed no or few policies to limit greenhouse gas emission and was presented with a lower, best and upper estimate. The other three ‘accelerated policy’ scenarios described future climates after emission reduction. In the second assessment report, the BaU scenario was elaborated in the IS92 scenarios (IPCC, 1995). Dutch researchers developed the global model IMAGE for impact assessment and policy development regarding greenhouse gases (Rotmans, 1990; Alcamo et al., 1999).

In this period, the first studies on climate and water appeared in the Netherlands. In a coastal defense study three sea level rise scenarios were considered, namely: the ‘policy’ scenario including sea level after global implementation of climate change mitigation policies; the ‘anticipatory’ scenario describing the best guess; and the ‘unfavourable’ scenario describing the best guess plus standard deviation (De Ronde and Vogel, 1988). Based on these scenarios, the subsequent ISOS (Impact of Sea level rise On Society) study quantified impacts, and identified policy options (Rijkswaterstaat and Delft Hydraulics, 1988). The study focused on safety against flooding, using scenarios on sea level rise, river discharges, wind and tidal conditions. The ISOS study was the first to include changes in river discharges in the scenarios. Socio-economic developments were excluded because of their uncertainty.

Now that safety and water supply were managed well, the government shifted its focus to water quality because: *‘pollution, together with overexploitation of water and an unbalanced spatial planning have resulted in an unsustainable water system’* (Rijkswaterstaat, 1988, p. 5). Accordingly, the 3rd PWM, entitled ‘Water for now and the future’, focused on ecological and chemical water quality provided that safety was guaranteed. The Brundtland report (Brundtland, 1987), which put sustainability high on the international political and public agenda, clearly inspired this quality focus. Policymakers defined future targets based on past conditions, and identified policy options to reach these target conditions under different scenarios. The scenarios included extrapolations of ongoing water demand trends and the intended result of environmental policy defined by the Ministry of the Environment. Although this ministry published three estimates, only the central estimate was considered.

While research studies extended their scope by using integrated scenarios, policymakers were focusing on safety issues. Triggered by the 1993 and 1995 flood events and the increased attention to climate change and sea level rise, the Dutch government installed the committee Tielrooy to analyse whether current water management was sufficiently prepared for future climate change and sea level rise. This committee adopted three of the KNMI1999 scenarios, which were similar to the KNMI1997 scenarios, but ignored the ‘dry’ scenario, because this scenario contained complementary signals compared to the other scenarios (wetter and warmer, drier and warmer, drier and colder). Socio-economic developments were only considered in a qualitative sense. In the final report, guiding principles to prepare for climate change were explicitly put forward: *‘anticipate instead of react, create more room for water, and do not only discharge, but also store water’* (CW21, 2000). As an alternative for confining water in narrow zones between dikes, creating more room for water was an upcoming paradigm in river management, aiming at decreasing water levels in times of peak discharges, and enhancing nature's quality at the same time (Dienst Landelijk Gebied, 1999; Silva et al., 2000). Regarding coastal zone management, the government decided in 2000 to double the amount of sand for beach nourishment in response to new insights on long-term morphological developments (Rijkswaterstaat and IMAU, 2000).

In 2003, several governmental organisations agreed in a so-called National Water Agreement (NWA) to define and implement strategies for coping with climate change and sea level rise by 2015, and to explore the necessary strategies for 2050 (Ministerie van Verkeer en Waterstaat, 2003). Water boards should adopt the guiding principles of the committee Tielrooy, and *‘at least use their central estimate scenario for 2050 with an outlook to 2100 to develop measures’*.

Until this period, policymakers neglected ‘drought’ as a possible effect of climate change. In 2002, the government studied the balance between fresh water demand and supply (RIZA, 2005). The dry summer of 2003 was a welcome surprise for getting the subject on the political agenda. KNMI updated the 1999 scenarios and re-introduced a ‘dry’ scenario in a revised version based on RCM results (Beersma, 2001). For the analysis also land use changes were included as well.

### 3.4 New climate scenarios and adaptation policy in legislation (2006 to present)

Based on extended and improved information of amongst others the IPCC's fourth assessment (IPCC, 2007), KNMI developed new climate scenarios; KNMI’06 scenarios (Van den Hurk et al., 2007; Katsman et al., 2008). As uncertainty due to emission scenarios was smaller than the uncertainty due to climate models, temperature was used as discriminating factor. A second relevant factor was the circulation regime. This resulted two scenarios with a moderate temperature increase (+1°C) and two with strong temperature increase (+2°C), which were further distinguished by a strong or weak change of atmospheric circulation over Europe. For sea level rise a bandwidth was given to cover the large variety in the sea level rises predicted by different climate models for different global warming scenarios. The four KNMI’06 scenarios were a problem for the water managers as this precludes the selection of a central estimate, as was prescribed in the NWA of 2003, and the adequacy of designed policy options needed to be reconsidered. The NWA was updated in 2008, and prescribed for different water related problems the use of only one of the KNMI’06 scenarios (Ministerie van Verkeer en Waterstaat, 2008). In 2009, KNMI reflected on the KNMI’06 report based on new scientific understanding and recent observations (Klein Tank and Lenderink, 2009). Although KNMI did not see the need for defining new scenarios, the scenarios with the moderate temperature changes were now considered less plausible than those with the larger changes. Consequently, again the guidelines in the NWA (Ministerie van Verkeer en Waterstaat, 2008) was outdated. For example, for studies on drought the NWA prescribed to use the ‘moderate dry’ scenario, while according to the update of KNMI for this kind of situations the ‘stronger dry’ scenario would be more plausible.

In 2007, the government established the second Delta committee for identifying actions to prevent future disasters (Kabat et al., 2009; Delta Committee, 2008), as the expected future climate change and sea level rise *‘can no longer be ignored’* (Delta Committee, 2008, p. 5). Next to the KNMI’06 scenarios, the committee considered a high-end scenario existing of a plausible upper limit of sea level rises in 2100 and 2200 for a robustness test of policies and investments (Katsman et al., 2011; Vellinga et al., 2008). The high-end scenario learnt policymakers that the Netherlands can overcome sea level rise and climate change, but that the water system has to be adapted. The advice resulted in a Delta Act and is presently being elaborated on in the so-called Delta Programme.

Climate change and sea level rise were now on the political and public agenda. In the 5th PWM (Rijkswaterstaat, 2009) climate change and sea level rise played an important role. The report had a separate chapter about dealing with uncertainties on climate change. The four KNMI’06 scenarios were described in detail, while socio-economic trends and future targets were described qualitatively. Again a scenario was prescribed for strategy development, meaning that the system should be prepared for coping with the situation described in a specific scenario. The report stated, that *‘For the choice of a scenario the societal risk is important. For safety issues the risk is larger, than for drainage and water logging issues. In case of low flexibility and high societal risk, there is a preference for the upper limits of climate change.’* (Rijkswaterstaat, 2009, p. 28). The report mentions the difficulties of including new scientific information: *‘The availability of repeatedly new scenarios results in the risk that decisionmaking will be postponed due to the uncertainties…On the one hand it is strived to use most recent insights while on the other hand stable assumptions are needed for decisionmaking and implementation. New insights cannot result in new assumptions and evaluations.’* (Rijkswaterstaat, 2009, p. 27). The report identified policy options to reach the described targets, and presented a planning scheme with research and decision milestones.

At European level, the Flood Directive (2007/60/EC) came into force in 2007. This directive aims at mapping and reducing flood risk and, as one of the measures, mapping flood-prone areas categorised to low, medium (likely return period ≥100 years), and high probability. The Flood Directive refers to these categories as scenarios. The 5th PWM states that it will incorporate this Directive in the Dutch legislation in the next planning period.

### 3.5 Dealing with uncertainties about the future: new approaches (2006 to present)

After 2000, the awareness raised that uncertainty over the future will remain and cannot be eliminated (cf. Van Asselt, 2000). More research does not automatically reduce uncertainty but may even increase it. Taleb (2007) emphasized future uncertainty with the introduction of the ‘Black Swans’ concept. These are unforeseen occurrences (unknown unknowns) with a low probability of occurrence but having a large impact. Although from a different field, the recent ‘economic crisis’ raised awareness that (unexpected) events influence our world view. New approaches for dealing with uncertainties emerged (e.g. Carter et al., 2007; Dessai and Hulme, 2004; Russill and Nyssa, 2009). Gladwell (2000) introduced the ‘tipping points’ concept to describe the catchiness of behaviour and ideas. Moser and Dilling (2007) used tipping points to conceptualise social change, and defined it as *‘moments in time where a normally stable or only gradually changing phenomena suddenly takes a radical turn.’* (Moser and Dilling, 2007, p. 492).

In the Netherlands, discussions on scenario updates led to a new approach, using the systems vulnerability to define Adaptation Tipping Points (ATP) indicating whether, and under what conditions, current water management strategies will continue to be effective under different climate changes (Kwadijk et al., 2010). In case of new scenarios, only the timing of an ATP needs to be updated. Events and surprises were recognised as triggers for adaptation, societal change and learning: not only the future endpoint, but also the pathway to this point is important. Therefore, a method to explore Adaptation Pathways was developed. By exploring pathways with transient scenarios, and including the dynamic interaction between the water system and society, policymakers can identify robust and flexible pathways or identify lock-ins (Haasnoot et al., 2011, in press; Offermans et al., 2011).

Also, at a policy level new concepts emerged. Recently, both the Scientific Council for Government Policy and the Advisory Council for the Ministry of Transport and Water Management advised to consider uncertainty explicitly (Van Asselt et al., 2010; Raad voor Verkeer en Waterstaat, 2009). The latter states that *‘we should not only be prepared for expected but uncertain future climates, but also for unknown uncertainties, so-called Black Swans.’* Accordingly, policy development should incorporate proactive adaptation by using scenarios for characterisation of uncertainties, and indicators to monitor the necessity of policy revision. The council also states that *‘policy based on an extreme scenario is liable to prove unduly expensive or unnecessary’* (p. 53). This statement is in contrast with the second Delta Committee. The scientific council requested attention for normative foresights including a variety of values and perspectives (Van Asselt et al., 2010).

The chair of the Delta Programme mentioned that: *‘One of the biggest challenges is dealing with uncertainties in the future climate, but also in population, economy and society. This requires a new way of planning, which we call adaptive delta planning. It seeks to maximise flexibility; keeping options open and avoiding ‘lock-in’* (Kuijken, 2011). These were starting points for a new approach for scenario design (Bruggeman et al., 2011). By analysing what makes policies for safety and water supply vulnerable, four climate and land use scenarios with small and large impact were established.

Originating from the 1990s, but becoming practice in the past years, is the paradigm shift occurring in the Netherlands from strategies of defence against water with hard engineering structures to a more ‘soft’ approach using natural dynamics of the system itself (cf. Inman, 2010). The changing approach involves restoration of wetlands, beaches and natural floodplains, and is referred to as ‘ecological engineering’, ‘building with nature’ or ‘green adaptation’ (e.g. Aarninkhof et al., 2010; Waterman, 2008; Van Koningsveld and Mulder, 2004). These approaches are novel ways of dealing with uncertainty: instead of fighting unpredictable future events, adapting to what is happening (Inman, 2010).

## 4 Key findings

### 4.1 Did the scenarios enable robust decision-making?

The central issue related to this question is whether the scenarios sufficiently represented relevant knowable uncertainties for enabling robust decisionmaking on water policies. We observed that scenarios in policy analysis shifted from describing future water *demand* to water *availability* after the 3rd PWM. For the 1st PWM policymakers expected no relevant changes in water availability. Research studies focused mainly on water availability scenarios in terms of climate change, sea level rise and river discharges. Thus, few studies included all relevant knowable uncertainties for long-term water management.

Whether the relevant uncertainties were *sufficiently* represented can be assessed from the number, value range, temporal and dynamic nature and the amount of alternatives. Over the past decades, the number of scenarios has increased from one to multiple scenarios, thereby increasing the represented uncertainty range. All research studies included several scenarios; first only climate scenarios, later studies also included socio-economic developments. The first policy documents considered a single scenario only, while policy studies in the past 15 years used three to four scenarios. Still, the guidelines for climate adaptation following from these policy documents recommended using only one scenario for the design of water policies (Ministerie van Verkeer en Waterstaat, 2003, 2008). Hence, although policymakers recognised uncertainty about the future with several scenarios, they persisted focusing on a ‘best estimate’ of the future climate in terms of a best prediction, until KNMI (deliberately) presented four scenarios in 2006 (Van den Hurk et al., 2007). Thereafter, policymakers selected one of these four scenarios as ‘best scenario’ for strategy development for a specific problem such as safety or water supply. Thus, in practise the range of the uncertainties was not fully considered.

Although an increasing number of scenarios was introduced, most scenarios remained to be extrapolations of trends. This is reflected by the scenario names. The first four policy documents merely used ‘business-as-usual’ scenarios called ‘trend’, ‘autonomous developments’ and ‘prognoses’. Few policy studies included a ‘maximum trend’, ‘worse case’ scenario. Only a few background studies tried to include alternatives, such as the ‘discontinuity’ scenario for the 4th PWM. In contrast, research studies explored more alternatives by considering several scenarios such as ‘worse case’, ‘lower/central/upper’ estimates, ‘dry’ and ‘cooling’ scenarios.

The dynamic and temporal nature of the scenarios were limited to defining a few projection horizons, in most cases the years 2050 and 2100. Scenarios described for these years were projections of climate and external context, resulting in a snapshot of the future situation beyond control of the water managers. Likewise, socio-economic drivers of water demand were considered as independent ‘policy driven’ or ‘autonomous developments’, which were gradual extrapolations of trends into the future. Adaptation options were then formulated and evaluated against external conditions at one future point. Scenario analysis for water management was, thus, a one-way pressure-impact analysis without response from society or water management, unlike global models, such as IMAGE (Rotmans, 1990). As a result, the water policy studies have ignored the dynamic path into the future with natural (year-to-year) variability, extreme events, the potentially large role of societal response to climate events and water management response to climate-associated events or changing socio-economic perspectives. It is only in recent scientific studies that this interaction is recognized, and that scenarios are becoming completed with these new relevant dimensions of time-series, dynamic interaction and surprises (Haasnoot et al., in press).

**Fig. 2:**
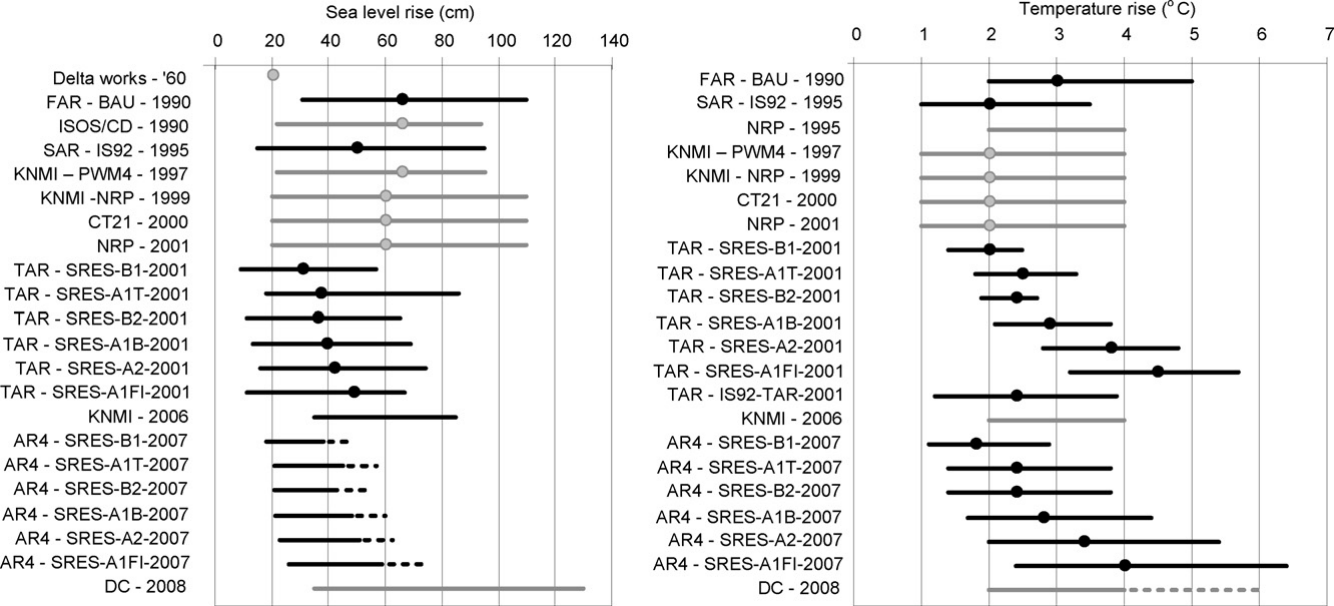
Values for global and local sea level rise for the Netherlands (left) and global temperature change (right) in 2100 for national and global climate scenarios (reference year 1990). FAR, SAR, TAR and AR4 refer respectively to the 1st, 2nd, 3rd and 4th IPCC report, NRP is National Research Programme, CT21 = Committee Tielrooy, DC = second Delta Committee, and PWM = National Policy Memorandum on Water Management. Scenarios for the Netherlands are in grey. In the DC study, the global temperature range included for the sea level rise was larger (dashed line) than for the climate parameters such as precipitation (solid line). In the AR4 report sea level rise values were presented for the scenarios (solid line), and additional uncertain sea level rise was described in the report (dashed line).

**Fig. 3:**
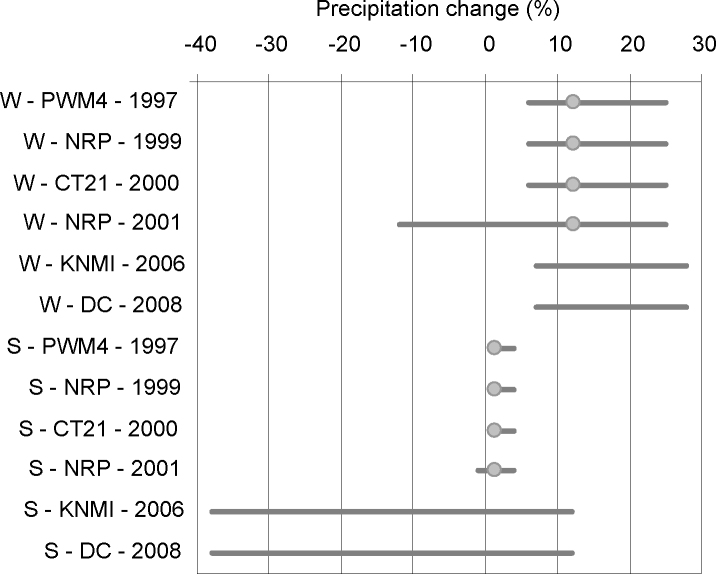
Values for precipitation change (w = winter; s = summer) in 2100 for different national climate scenarios. PWM = National Policy Memorandum on Water Management, NRP is National Research Programme, CT21 = Committee Tielrooy, and DC = second Delta Committee.

The range of the values used in the scenarios is an additional indicator for the sufficient representation of uncertainty (see Figs. 2 and 3 for climate scenarios and supplementary information for socio-economic developments). The 1st and 2nd PWM used one value based on trends for water demand, but extended the range due to climate variability by analysing years with different net precipitation and discharge. Three studies translated socio-economic developments into land use maps. The projection year of these scenarios extended from 2015 to 2050 to 2100 resulting in an increase of the considered acreage change and the bandwidth for urban and nature, but not for agriculture. Regarding the climate scenarios, the bandwidth of the emission and global temperature changes in the IPCC scenarios has become larger. Previous climate scenarios for the Netherlands had similar ranges for the global temperature as the IPCC scenarios, but recent scenarios differ from the IPCC assessments. The bandwidth for global temperature rise used in the Netherlands (Fig. 2) is remarkably smaller than the IPCC scenarios at that time. This is caused by the fact that the KNMI scenarios represent approximately 80% of the total range of the output of the climate models, while IPCC scenarios presented the complete range. However, it is uncertain whether water managers and the general public in the Netherlands are aware of this difference, and only see the smaller uncertainty range. Over the years, KNMI's scenario values for summer precipitation have changed considerably, in contrast to the winter values. The introduction of the ‘dry’ scenarios reflects the awareness of larger uncertainty about future summer climate, as not only the magnitude, but also direction of the change differed in the scenarios.

The difference in projections of sea level rise between IPCC and the Dutch scenarios is striking (Fig. 2). While the IPCC scenarios show a trend to narrower ranges and smaller values for sea level rise, the KNMI kept the same range and the values were larger than the IPCC. These differences can mainly be explained from the different uncertainties included in the scenarios (e.g. the uncertainty in the contribution of ice sheets). In the AR4 study part of the uncertainties related to ice sheets was not included in the sea level scenario values, but only described in the report. These uncertainties were, however, included in the national KNMI scenarios, together with recent (scenario and field) studies which were not available at the time of the AR4 (Katsman et al., 2011). In addition, regional differences due to variation in ocean temperature, distribution of melt water over the oceans, and – in some studies – tectonic subsidence contribute to differences between the scenario studies. For example, in the 1990s studies values were derived from the IPCC estimates, supplemented with the natural trend and subsidence of the Netherlands (Van Asselt et al., 9582). The Delta Committee included a tectonic subsidence of 10 cm/year (Vellinga et al., 2008), while the studies in the 1990s included a subsidence of 5 cm/year. The high-end sea level rise explored by the 2nd Delta Committee was discussed thoroughly among researchers and policymakers. The values were larger than in the KNMI’06 scenarios, because the Delta Committee aimed at defining an ‘upper plausible’ limit of sea level rise by including a wider range of uncertainties and mechanisms underlying sea level rise for the Netherlands. Remarkably, this upper level is not that much higher than the upper ends of the uncertainty ranges put forward in 1990 in the national studies.

### 4.2 Did the scenarios enable learning?

Generally, scenario analysis in water policy studies enabled four different lessons: (1) insight in impacts of climate change and socio-economic developments, as a result of several national, but also global studies (e.g. IPCC reports, ISOS and NRP studies); (2) the need and effectiveness of policies, such the 2nd PWM or the ATP study; (3) the need for adaptation of targets and/or policies as a result of comparing scenarios with monitoring results (e.g. 2nd and 3rd PWM); and (4) awareness about possible impacts of climate and socio-economic developments. For example, the second Delta Committee widely communicated its results through readable reports and YouTube videos accessible for the general public. This received a lot of media attention, and raised the awareness of the importance for developing water management strategies to prepare for the future. Furthermore, their ‘worst case’ scenario deliberately provoked lots of discussion among water managers in the Netherlands, which enhanced the exchange of ideas, and thus involved a large degree of learning according to the chair of the committee (Veerman, 2010). Flood and drought events corresponding with the scenarios, but also the public debate about issues (e.g. climate change, credit crisis) accelerated the influence of study results in policy implementation.

Both scenario analysis in water management and the science-policy interaction have clearly evolved in the past twenty years. In retrospective we can distinguish five evolutions that reflect the learning process of scientists and policymakers:

1. *From flood protection to integrated water management*: This shift was supported by lessons on the effectiveness of policies in scenario analysis. After the major flooding of 1953, water management focused on flood protection. However, in the course of time, and with the step-wise completion of the Delta works, attention was given to other water-related problems. In the PWMs the focus changed from water supply for economic purposes, via a cost–benefit analysis for maintaining water availability to water quality and nature, and eventually introducing the concept of ‘integrated water management’, which the 5th PWM extended with spatial planning issues. Also, the scientific studies show a learning process through an evolution in the studied subjects. The first research studies focused on safety against coastal flooding, which was later extended to large rivers and regional water systems and finally to impact assessments of water services.
2. *Towards integrated scenarios*: This shift was initiated by awareness that both water *availability* and water *demand* are relevant for water policymaking, as well as the global and European shift to integrated studies. Also, scenario studies showed the relevance of integrated studies for decisionmaking. Although coming from a different starting point, both scientific and policy studies moved towards integrated scenarios. Scientific studies first used climate scenarios. By the end of the 1990s, socio-economic developments were considered increasingly relevant. After only evaluating land use change trends and ‘autonomous’ socio-economic developments, integrated scenarios comprising both climate and socio-economic components were defined to explore different water management styles. The scenario content in the PWMs changed in correspondence with the purpose of the PWMs from water demand trends to climate scenarios, while at present integrated scenarios are considered. Still, the integrated scenarios are not yet fully employed for impact assessment or policy development. Furthermore, the influence of societal perspectives (e.g. on policy targets) remains to be fully incorporated in policymaking.
3. *From predicting to exploring the future*: While policymakers experienced that the future turned out differently than envisioned, and some events occurred as complete surprise, evidence grew that we cannot predict the future. Initially, prognoses only applied to possible changes in water demand. Estimates of future flood magnitudes – as required for the probabilistic flood protection approach – were based on autonomous developments or expert judgement. These ‘predict and act’ studies slowly shifted to an ‘explore and anticipate’ approach for which several scenarios were used. Still, the initial use of ‘best guess’ or ‘central estimate’ climate scenarios reflects the desire of predicting future conditions, although now associated with bands of uncertainty. With the IPCC-SRES and KNMI’06 scenarios, the recognition that the future is uncertain and that there is no ‘most likely’ future, has increasingly settled in water management. Accordingly, research and policy studies not only aimed at improving the understanding of future developments such as climate change and reducing uncertainties, but also on developing methods for dealing with uncertainties about the future. This observed shift corresponds with observations of futurists (Van Asselt et al., 2010; Slaughter, 2002; Van t Klooster, 2008). Both approaches, also referred to as forecasting and foresight, are still used next to each other (Van Asselt et al., 2010). Also, in water management the predictive approach is still used when it comes to short term actions such as flood forecasting and determining the (long-term) design discharge. For short term drought management both forecasts and scenarios (foresights) are used. Some analysts propose to use probabilistic scenarios, but we have not observed these scenarios in the studies reviewed, but this could be initiated by the EU Flood Directive's approach, which prescribes to use scenarios with floods with low, medium and high probability.
4. *Interaction science, policy and events*: Most uncertainties about the future were first investigated by scientists, and later incorporated in policy, especially if events seemed to support the trends indicated by scenarios. For example, the 3rd and 4th PWM documents mentioned potentially relevant impacts of climate based IPCC results and scientific research in the preceding decades. In recent years, the turn-over rate from scientific studies to water management has speeded-up. Scientific studies involve stakeholders and while novel approaches in scenario analysis emerge briefly after being introduced in the scientific world in water management approaches as well.
5. *From fighting water to accommodating and adapting to water*: Since the 1960, awareness raise about potential effects of climate change as a result of scenario studies, and flood events. This awareness triggered a shift from focusing on ‘hard’ defensive infrastructures for flood protection to ‘softer’ measures for integrated water management, by using natural processes and accommodating water (e.g. 4th PWM). Thus, instead of static infrastructures with a long life time, easily adaptable policies to changing, unpredictable boundary conditions were chosen.

## 5 Conclusions and recommendations

This review describes the use of scenarios in water management studies in the Netherlands over the past 60 years. To identify what we have learnt from this experience, we analysed whether the scenarios enabled robust decisionmaking and learning.

The opportunities for robust decisionmaking resulting from scenarios increased, but are still not fully exploited, especially in policymaking. Although the number of scenarios increased, for the strategy development often one scenario was appointed for design conditions. Rarely, all relevant uncertainties were included. Especially in the policy documents uncertainties in water *demand* or *availability* were considered, while none included social (perspective-based) uncertainty. The number of alternative futures increased, but scenarios mainly remained based on extrapolation of trends. Almost all scenarios used were snapshots at 2 or 3 time horizons, thereby ignoring pathways towards the endpoint, and disregarding the possibility that events may drastically change such pathways. All scenarios were surprise free. The ‘decision robustness’ can thus be improved.

Differences in value range between different scenario studies can often be explained by reading details and communicating with the developers, which indicates that communication on assumptions is important for appropriate scenario use.

The scenarios enabled learning about possible impacts of developments, the need and effectiveness of policies, and the need for adaptation of policies. In addition, the scenarios raised awareness about potential future problems. The historical perspective shows a clear science-policy interaction. For example, first used in research studies, the policy documents took climate change and sea level rise up, as important developments to consider in strategy development; sometimes with a little help of a flood or drought event. We observed several paradigm shifts reflecting the learning process of scientists and policymakers: (a) from flood control to integrated water management, (b) from predicting to exploring the future with integrated scenarios, and (c) from fighting water to accommodating and adapting to water.

Dealing with uncertainties appears to be a struggle, given the paradox between the desire to explore potential futures using several different scenarios, *and* the preference of water managers to design policies based on a single scenario that is not frequently updated. However, water managers need to face that the future is inherently uncertain, and scenarios are always likely to be updated by new scenarios as they result from a process of design and construction at a specific moment and location (Hulme and Dessai, 2008b). These uncertainties should not be used as a constraint to develop adaptation measures for water management (cf. Dessai et al., 2009; Hulme and Dessai, 2008b).

We provide five recommendations for improving water policy development under uncertainty:

1. For sustainable decisionmaking water managers should consider several scenarios to explore the relevant range of the uncertainties, and not selecting the most likely future or prescribing a ‘design’ scenario.
2. New approaches are available, which can together with scenario analysis support the development of sustainable measures. Several methods involve many computational experiments to analyse the effects of uncertain parameters (e.g. ‘Exploratory Modeling’ Bankes (1993)) to seek for robust decisions (Lempert et al., 2006; Lempert and Bankes, 2003) or optimal solutions (‘Info Gap’ theory Ben-Haim (2001)). Walker et al. (2001) describe a planning process with different types of actions (e.g. ‘mitigating actions’, ‘hedging actions’) and signposts to monitor if adaptation is needed. Also, adaptation tipping points (Kwadijk et al., 2010) and exploring adaptation pathways with transient scenarios (Haasnoot et al., 2011) can be of assistance.
3. Scenario developers should clearly communicate the assumptions, purpose and limitations of scenarios, and the conditions under which the scenarios were made (process and time limits).
4. Tailored scenarios are needed to ensure relevant scenarios and appropriate use. To develop tailored scenarios water managers should assess the system's vulnerability and communicate this to scenario developers.
5. To improve scenarios and their use, evaluation of past scenarios remains useful. For this purpose, evaluation on ‘Decision robustness’ and ‘Learning success’ deserve further elaboration in terms of more explicit criteria concerning e.g. comparison with study's objectives, stakeholder involvement, pathway analysis, more precise addressing of the learning effect (who learned what and how?).
6. Instead of responding to flood and drought events, policymakers could identify triggers (Walker et al., 2001) and adaptation pathways (Haasnoot et al., 2011). The triggers give signals when it is time to make a decision and the adaptation pathways allow for identifying robust options and lock-ins.

Summarizing, exploring the future with several scenarios, analysing the vulnerability and good communication with scenario developers may help water managers to deal with uncertainties, and make sustainable decisions.
