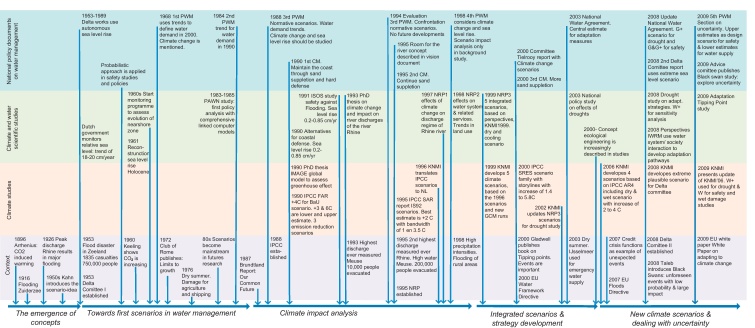# Erratum to “A history of futures: A review of scenario use in water policy studies in the Netherlands” [Environ. Sci. Policy 19–20 (2012) 108–120]

**DOI:** 10.1016/j.envsci.2012.04.004

**Published:** 2012-05

**Authors:** M. Haasnoot, H. Middelkoop

**Affiliations:** a Deltares, P.O. Box 177, 2600 MH Delft, The Netherlands; b Utrecht University, Department of Physical Geography, P.O. Box 80115, 3508 TC Utrecht, The Netherlands; c University of Twente, Department of Water Engineering and Management, P.O. Box 217, 7500 AE Enschede, The Netherlands

The publisher regrets that Fig. 1 was not published completely. The correct version of Fig. 1 is presented below.

The publisher would like to apologise for any inconvenience caused.

**Figure:**