# Supplementary material for: A history of futures: A review of scenario use in water policy studies in the Netherlands
Source: Environ Sci Policy. 2012 May;19-20(6):108–20. doi: 10.1016/j.envsci.2012.03.002 (PMC3587454; doi:10.1016/j.envsci.2012.03.002)
Supplement: Supplementary file 1 [file mmc1.doc]

**Supplementary information**

**Characteristics of scenarios**

Characteristics of scenarios used in the national policy documents (National Policy Memorandum on Water Management, PWM) and research studies on climate and water. Next section gives a short explanation of the scenario typology used to describe the characteristics.

| **Name study** | **Year & reference** | **Purpose study** | **Context of study** | **Number** | **Type scenario** | **Quantitative / qualitative** | **Alternative futures** | **Time horizon** | **Name**  **scenarios** | **Variables in the**  **scenarios** | **How is uncertainty**  **mentioned** | **Results of**  **study** | **Policy / research** |
| --- | --- | --- | --- | --- | --- | --- | --- | --- | --- | --- | --- | --- | --- |
| Delta works | 1950-1960 | Safety against coastal flooding | 1953 storm surge resulted in numerous casualties and large scale flooding of south-western part of the NL. | 1 | Predictive - forecast | Quantitative | Small | +100 & +200 | No information available | Sea level rise | No information available | Policy options implemented | Policy |
| 1^st^ PWM | 1968 | Improve water supply | The Delta works were partly finished. Safety was ok. Increasing population and intensive use space industrialisation and more navigation result in higher demands on water (quantity and quality). | 1 | Predictive - forecast | Quantitative | Small | 2000 | Referred to as trends or prognoses | Water demand for agriculture, drinking and industry water. Climate variability is considered by analysing 2 different ‘dry’ years | Uncertainty about future water demand was acknowledged but no bandwidth given. Impact of uncertainties in upstream developments were considered small. Climate change, sea level rise and upstream developments influencing river Rhine discharge were mentioned not included.. | Policy options defined and implemented. No additional policy options due to future developments | Policy |
| 2^nd^ PWM | 1984 | Improvement water management, cost/benefit analysis | "Prognoses" on water demand needed to be revised. Also industry, shipping and nature were acknowledged as water users. | 1 | Predictive - forecast | Quantitative | Small | 1976-1990 | Reference/no policy, prognoses, maximum trend sprinkling scenario | Water demands for agriculture and drinking and industry water. Maximum trend (high growth industry and high sprinkling scenario). Climate variability is considered by analysing 5 different ‘dry’ years | Uncertainty about future and working of system were included by sensitivity analysis or scenarios. 1990 was chosen as future year because small uncertainties about developments | No extra policy options needed | Policy |
| PAWN study. Background study 2^nd^ PWM | 1985 | Provide insight in national water system in the Netherlands, assess potential problem and solutions | "Prognoses" on water demand needed to be revised. Also industry, shipping and nature were acknowledged as water users. | 1 | Predictive - forecast | Quantitative | Small | 1976-1985/1990 | Reference/no policy, prognoses, low and high sprinkling scenarios | Water demands for agriculture and drinking and industry water. Climate variability is considered by analysing 5 different ‘dry’ years | Uncertainty about future and working of system were included by sensitivity analysis or scenarios. 1990 was chosen as future year because small uncertainties about developments | No extra policy options needed | Policy/ Research |
| Discussion report Coastal defense | 1988 | Safety against flooding: impact and policy options | Jelgersma published curve on sea level rise. Establishment IPCC. | 3 | Explorative - external | Quantitative | Large | 2050, 2100 | Policy (autonomous developments), anticipatory (best guess), unfavourable | Sea level rise, wind power, tidal range | Sensitivity and scenario analysis. The chance that the future would be more unfavourable than worse case was estimated at 5 to 15 %. | Impact assessment | Research |
| ISOS | 1988 | Safety against flooding: impact and policy options | Jelgersma published curve on sea level rise. Establishment IPCC. | 3 | Explorative - external | Quantitative | Large | 2050, 2100 | Autonomous developments, best guess, unfavourable | Sea level rise, river discharges, wind power, tidal range | Sensitivity and scenario analysis was executed. Socio-economic developments were considered to be too uncertain to anticipate on. The chance that the future situation would be worse than the unfavourable scenario was estimated at 5 a 15%. River discharges were estimated (-5 and -10% in summer and +5 and +10% in winter. | Policy options implemented | Research |
| 3^rd^ PWM | 1994 | Implementation integrated water management. To achieve long-term objectives | Brundtland report on sustainability was published. Upcoming concern of the chemical and ecological quality of the rivers | 1 | Predictive-forecast, Normative | Quantitative and qualitative | Small (BAU) | not mentioned | Trends, target conditions, central estimate Environmental policy | Ecological normative scenarios. Alternative policy strategies. Trends agricultural water use, drinking water use, emissions and electricity production. | Some developments may make it difficult to achieve objectives. In that case objective may need to be adjusted. Impacts of climate change and sea level rise should be further investigated. Demand of society may change. | Policy options identified | Policy |
| Aquatic Outlook  Background study 4th PWM. | 1996 | Define policy options to make the water system more resilient. Assess target conditions considering soci-economic developments | Brundtland report and the need for systematic quantitative analysis on how to achieve targets of 3^rd^ PWM. Upcoming concern of the chemical and ecological quality of the rivers. | 2 & 4 policies | Predictive - what-if | Quantitative and qualitative | Small for external scenarios and large for policies | 2015, 2045 | Land use: European Renaissance and Balanced Growth  Policy: Business as Usual (Current Policy) 2015, Use Policy 2015, System Policy 2015, Radical change (Discontinuity) Policy 2045 | Land use changes from socio-economic scenarios and policy options. Current climate conditions in terms of an average year and average river discharge were used | In case relevant, a sensitivity analysis was done for climate change and economic scenarios, including sea level rise and its impact on the lake IJsselmeer and an increase of the design discharge of the river Rhine. The document clearly stated that the strategy scenarios should be used as different policy options and elements for the final water management strategy in the 4th PWM. | Policy options | Policy |
| NRP1 | 1997 | Impact assessment on discharge river Rhine | National Research Program on climate change. | 2 | Predictive - what-if | Quantitative | Small | 2050, 2100 | UKHI, XCCC (after GCM) | Temperature, evaporation wind, radiation | The bandwidth of the results is wide and is primarily caused by uncertainty in climate scenarios. Also, downscaling GCM and model variety play a role. Evaporation (influence of CO2 and biomass increase) is major uncertainty for low flows. | Impact assessment | Research |
| 4^th^ PWM | 1998 | Safety and sustainable water systems with good water quality | High waters at river Rhine & Meuse in '93 & '95 pointed out the vulnerability of living in a lowing lying river delta. Without policies future flood risk will increase due to climate change . | 1 | Predictive-forecast, Normative | Quantitative and qualitative | Small (BAU) | not mentioned | Prognoses, target conditions | Normative scenarios per water system and theme and prognoses for water demand for shipping, drinking & industry water (no agriculture) | There is uncertainty about effects of climate and sea level rise, more research is needed. Need to incorporate room for uncertain and unforeseen developments was expressed. | Guiding principles for policy options | Policy |
| NRP2 | 1999 | Impact assessment water management in Netherlands | National Research Program on climate change. | 3 | Predictive - what-if | Quantitative | Moderate | 2050, 2100 | Reference (2050,2100), Lower, central, upper estimation | Temperature, precipitation, evaporation, sea level rise, land use (Netherlands & Rhine Upstream) | It was explicitly mentioned that scenarios are not predictions, but they were considered as a plausible basis for a what-if sensitivity analysis | Impact assessment and policy options identified | Research |
| Comittee Tielrooy | 2000 | Prepare for climate change and sea level rise | High waters at river Rhine & Meuse, evacuation of people from floodplain areas in '93 & '95, flooding regional areas '98. | 3 | Predictive - forecast | Quantitative and qualitative | Small | 2030 for socio- economic 2050, 2100 for climate | Minimum, central, maximum scenario | Temperature, precipitation (year, summer, winter, rain), evaporation, sea level rise, river discharge (design, summer, winter). | The lower estimate will probably occur in the current trend, thus without greenhouse effect. Effects such as river discharge and sea level rise are presented as effects that will occur, with a maximum degree given by the maximum scenario | Guiding principles for policy options | Policy |
| NRP3 | 2001 | Development of water management strategies. Method for integrated scenario analysis | High waters at river Rhine & Meuse, evacuation of people from floodplain areas in '93 & '95, flooding regional areas '98. IPCC report concluding global warming trend has been occurring over past century. | 5 | Explorative - external | Quantitative and qualitative | Large | 2050, 2100 | Lower, central, upper wet, upper dry, nao change | Temperature, precipitation, evaporation, sea level rise, land use, perspective | Include uncertainty about climate, socio-economic developments and perspectives | Impact assessment and policy options | Research |
| National Water Agreement | 2003 | Give guidance and norms for adaptation for regional governments | Results of committee Tielrooy needed to be translated in legislation (norms). | 1 | Predictive-forecast | Quantitative | Small | 2050 | Central estimation | Refer to Committee Tielrooy | Anticipate to climate change for at least the central estimate of the Tielrooy committee in 2050 | Legislation | Policy |
| Drought study | 2003 | Impact assessment drought and identification of strategies | Awareness that water management focused a lot on wet conditions. Dry summer 2003. Dike breach of peat dike due to drought resulted in flooding. | 3 | Predictive-forecast | Quantitative | Large | 2050 | Lower (individualist), central (hierarchist), upper wet (egalitarian), upper (dry), change in transpiration, sensitivity analysis for land use change. | a.o. temperature, precipitation (year, summer, winter), evaporation, sea level rise, river discharge. Climate variability is considered by analysing 5 different ‘dry’ years. | Effects are presented in possibilities and a bandwidth is given. The bandwidth is a result of different scenarios. The relation is with these scenarios not always given. Sensitivity analysis was for effects of transpiration due to CO2 increase | Identification of strategies | Research |
| Drought management study | 2008 | Impact assessment drought and identification of strategies, update of previous drought study | New KNMI scenarios in 2006. | 1 | Possible - external | Quantitative | Small | 2050 | KNMI'06: moderate+, warm+ | a.o. temperature, precipitation (year, summer, winter), evaporation, sea level rise, river discharge. Climate variability is considered by analysing 5 different ‘dry’ years. | Most extreme KNMI'06 scenario was taken to explore policy options using the following assumption: if it is not profitable to take measure under this scenarios, there is no reason to implement policies. Climate variability analysed by looking at characteristic years | Identification of strategies | Research |
| Perspectives IWRM | 2008 | Develop method to deal with uncertainty about the future | Pathway and interaction between society and water system is considered as important. | many | Explorative - external | Quantitative and qualitative | Large | chain, until 2100 | Transient (time-series) scenarios based on KNMI'06, socio-economic scenarios | a.o. temperature, precipitation, river discharges, sea level rise, land use, perspectives. | Include uncertainty about climate, socio-economic developments and perspectives. Also interaction between society and water system | Method for decision making, ongoing project | Research |
| Update National Water Agreement | 2008 | Give guidance for adaptation for regional governments | New KNMI scenarios in 2006. | 2 | Predictive - forecast | Quantitative | Small | 2050 | KNMI'06: moderate+, warm+ | Refer to KNMI'06 | For tasks already considered the central estimate corresponding with the KNMI'06-moderate scenario should be used. If financial possible, policy options should be implemented given the uncertainties and extreme of KNMI'06 scenarios | Legislation with prescription of design conditions | Policy |
| Committee Veerman | 2008 | Chart a course of action to prevent future disasters and to raise awareness of the importance to develop strategies | Raising awareness on potential impacts of climate change and sea level rise. | 4 | Explorative - external | Quantitative and qualitative | Large | 2050, 2100, 2200 | Plausible upper limit (sea level), KNMI'06: moderate+, warm+ | Refer to KNMI'06. Socio-economic trends qualitatively | There is still a lot of uncertainty about the extent and velocity of climate change and its effects. Use plausible upper limits of climate change to avoid that future generations will be confronted with unforeseen worse effects | Awareness, legislation, research program | Policy |
| 5^th^ PWM | 2009 | A safe and liveable delta now and in the future | Delta Committee | 5 | Normative - transforming, Predictive - forecast | Quantitative and qualitative | Large | several 2050, 2100 | KNMI'06: moderate+, warm+; trends (socio-economic); plausible upper limit (sea level) | a.o. sea level, temperature, precipitation, number of wet days, wind velocity for winter and summer. Socio-economic and demographic trends are mentioned qualitatively | Uncertainty is explicitly mentioned in separate chapter. Develop robust and flexible strategies to deal with uncertainties about the future. | Guiding principles and roadmaps for decision making | Policy |
| Adaptation Tipping Point | 2009 | Explore vulnerability water management for climate change. Develop method to deal with uncertainty | Discussion at water boards on how to deal with 4 climate scenarios as central estimate can be chosen as norm. | 4 | Explorative - external | Quantitative | Large | chain, until 2100 | KNMI'06: moderate+, warm+ | Focus on sea level rise and river discharges | Assess vulnerability and use possible scenarios to determining timing of tipping point. | Method and indication of vulnerability | Research |
| Delta-scenarios for Delta  Programme | 2010, ongoing | Define policy options to prepare for climate change and sea level rise | Advice of Delta Committee | 4 | Explorative - external | Quantitative | Large | 2050, 2100 | Pressure, Steam, Warm, Quiet | a.o. temperature, precipitation, sea level, river discharge, population, economic growth, land use (urban, agriculture, nature). , Based on KNMI’06 and WLO socio-economic scenarios | Refer to two reports (Raad voor Verkeer en Waterstaat 2009 and Van Asselt 2010) which advice to include uncertainty explicity in policy analysis | Ongoing | Policy |

**Values for socio-economic developments in the scenarios**

|  | 1st PWM | | 2nd PWM | | Aquatic Outlook background PWM4 (1996) | | | | |
| --- | --- | --- | --- | --- | --- | --- | --- | --- | --- |
| Scenario name | Prognoses | Prognoses | Prognoses | Prognoses | Current | Current Policy | Use Policy | System Policy | Radical change Policy |
| Projection year | 2000 | 2000 dry year april/sept. | 1990 dry year | 1990 extreme dry year |  | 2015 | 2015 | 2015 | 2045 |
| Urban area % of total area |  |  |  |  |  |  |  |  |  |
| Agriculture % of total area |  |  |  |  | 72 | 64 | 64 | 64 | 58 |
| Nature % of total area |  |  |  |  |  |  |  |  |  |
| Population (milj.) | 17.9 |  |  |  |  |  |  |  |  |
| Economic growth (%/year) |  |  |  |  |  |  |  |  |  |
| Drinking water demand houses (milj. m^3^/year) | 1100 | 600 | 1500 (incl. industry)\ | 1500 (incl. industry) |  |  |  |  |  |
| Maintaining water levels and irrigation agriculture milj.m^3^ in 95% dry year | 3300 | 3300 |  |  |  |  |  |  |  |
| Irrigation |  |  |  |  |  | -40% | -40% | -75% | -100% |
| Flushing / irrigation agriculture | 12200 | 6100 | 991/1509 | 1470/2769 |  |  |  |  |  |
| Industry | 5500 | 2700 | +300 drinking & +150 groundwater | +300 drinking & +150 groundwater |  |  |  |  |  |

|  | backgr. PWM4, NRP2 (1999) | | | | Drought study (2002) | | | | Delta Programme (2011) | | | |
| --- | --- | --- | --- | --- | --- | --- | --- | --- | --- | --- | --- | --- |
| Scenario name | Current | ER | BG | GS | Current | EGA | HIE | IND | Steam/Pressure | | Warm/Quiet | |
| Projection year |  | 2015 | 2015 | 2015 |  | 2050 | 2050 | 2050 | 2050 | 2100 | 2050 | 2100 |
| Urban area % of total area | 10 | 13 | 13 | 12 | 17 | 14 | 13 | 14 | 20 | 25 | 17 | 10 |
| Agriculture % of total area | 74 | 65 | 60 | 68 | 67 | 53 | 62 | 55 | 59 | 70 | 62 | 67 |
| Nature % of total area | 16 | 21 | 25 | 19 | 16 | 33 | 25 | 31 | 21 | 5 | 21 | 23 |
| Combination Agriculture/Nature % of total area |  | 1 | 3 | 1 |  |  |  |  |  |  |  |  |
| Population (milj.) |  |  |  |  |  | 14.6 | 16.4 | 18.9 | 20 | 24 | 15 | 12 |
| Economic growth (%/year) |  |  |  |  |  | 1.5 | 2.75 | 3.25 | 2.6 | 2.0-2.6 | 0.7 | 0-0.5 |
| Drinking water demand houses (milj m3/year) |  |  |  |  |  | 1500 | 1900 | 1900 | - | - | - | - |

**Scenario typologies**

In this paper we characterize the use of scenarios based on two scenario typologies. First of all, we follow van Notten et al. (2003) to describe ‘why’, ‘how’, ‘what’: what was the goal, the process design and the content of the scenarios? Regarding the goal of the scenario analysis they distinguish between normative and descriptive scenarios, which describe respectively preferable futures (including norms) and possible futures. Regarding the vantage point scenarios can be either forecasting, taking the present as a starting point, or backcasting, reasoning from future situation to explore paths to reach this situation. Qualitative versus quantitative scenarios is used as one of the characteristics for the process design. Qualitative scenarios are narratives, possibly developed together with stakeholders. Quantitative scenarios, frequently used in environmental studies, are often developed using computer simulations. Regarding the content of scenarios, Van Notten et al. use the temporal nature, nature of dynamics and the level of deviation as characteristics. Snapshot scenarios describe a moment in the future, while chain scenarios describe the evolvement to a certain point in the future. Scenarios can be either surprise free, often describing trends, or discontinuous, including events which change the developments abruptly. The level of deviation of scenarios refers to the extent to which alternative futures are described or whether only trends are considered.

The second typology we use distinguishes three categories for the classification of the scenario type, namely: ‘predictive’ (what will happen), ‘possible’ (what can happen?) and ‘normative’ (hoe can a target be reached?) scenarios after the typology of Borjeson et al. (2006). Within these three categories they further divide to achieve 6 types:

- forecasts scenarios: describe what will happen if the most likely development unfolds;
- what-if scenarios: are used to investigate what will happen on the condition of some specified near future events;
- external scenarios: focus on factors beyond the control of the relevant actors;
- strategic scenarios: incorporate policy measures of the intended scenario user;
- preserving scenarios: are used to find out how a certain target can be met; and
- transforming scenarios: are similar to the preserving scenarios but this target seems to be unreachable if the ongoing developments continue .
